# Foot function and strength of patients with diabetes grouped by ulcer risk classification (IWGDF)

**DOI:** 10.1186/s13098-019-0487-x

**Published:** 2019-10-30

**Authors:** Jane S. S. P. Ferreira, João P. Panighel, Érica Q. Silva, Renan L. Monteiro, Ronaldo H. Cruvinel Júnior, Isabel C. N. Sacco

**Affiliations:** 1 Department of Physical Therapy, Speech, and Occupational Therapy, School of Medicine, University of São Paulo, Rua Cipotânea, 51-Cidade Universitária, São Paulo, 05360-160 Brazil; 2 Departamento de Ciências da Saúde, Universidade Federal do Amapá, Macapá, Amapá Brazil

**Keywords:** Muscle weakness, Diabetic neuropathies, Diabetic foot, Ulcer

## Abstract

**Background:**

The stratification system from the International Working Group on the Diabetic Foot (IWGDF) was used to classify the participants as to the ulcer risk. However, it is not yet known what the classification groups’ individual deficits are regarding sensitivity, function, and musculoskeletal properties and mechanics. This makes it difficult to design proper ulcer prevention strategies for patients. Thus, this study aimed to investigate the foot function, foot strength and health of people with diabetes mellitus (DM)—with or without DPN—while considering the different ulcer risk classifications determined by the IWGDF.

**Methods:**

The subject pool comprised 72 people with DM, with and without DPN. The patients were divided into three groups: Group 0 (G0), which comprised diabetic patients without DPN; Group 1 (G1), which comprised patients with DPN; and Group 2 (G2), which comprised patients with DPN who had foot deformities. The health and foot function of the subjects’ feet were assessed using a foot health status questionnaire (FHSQ-BR) that investigated four domains: foot pain, foot function, footwear, and general foot health. The patients’ foot strength was evaluated using the maximum force under each subject’s hallux and toes on a pressure platform (emed q-100, Novel, Munich, Germany).

**Results:**

Moderate differences were found between G0 and G1 and G2 for the foot pain, foot function, general foot health, and footwear. There was also a small but significant difference between G0 and G2 in regards to hallux strength.

**Conclusion:**

Foot health, foot function and strength levels of people with DM and DPN classified by the ulcer risk are different and this must be taken into account when evaluating and developing treatment strategies for these patients.

## Background

Plantar ulcers present major challenges for people with diabetes mellitus (DM), with a prevalence ranging from 0.65% in individuals under 44 years old to 1.3% in individuals aged 75 years or older [1]; moreover, the ulcers have higher incidences in developing countries [2] and a 41.5% chance of reocurrence [3].

Amongst the most common chronic complications of diabetes are neuropathies, affecting up to 50% of DM patients [4]. The diabetic polyneuropathy (DPN) is characterized by loss of protective sensation, which in turn contributes to the appearance of plantar ulcers [4]. According to IWGDF, the loss of protective sensation can be assessed with one of the following techniques: pressure perception using a Semmes–Weinstein 10 g monofilament or vibration perception using a 128 Hz tuning fork. When a monofilament or a tuning fork are not available, it is recommended to test the tactile sensation by lightly touching the tips of the toes with the tip of the index finger for 1–2 s [5]. The IWGDF Risk Stratification System classifies people with diabetes in four groups according to the ulcer risk. Group 0, which comprised patients without DPN; Group 1, which comprised patients with DPN; Group 2, which comprised patients with DPN and with foot deformities and/or vascular disease; and Group 3, which comprised patients with DPN and history of a foot ulcer or a lower-extremity amputation (minor or major) or end-stage renal disease [5].

Sensitivity changes are due to DPN [6–10], which begins with impairments to peripheral nerves and progresses to impairments to motor and autonomic nerves. DPN results in progressive vibratory, thermal, tactile, and proprioceptive sensitivity deficits [10]. In addition to affecting the integrity of neural structures, DPN also affects musculoskeletal structures, such as lower limb joints, the calcaneal tendon, and intrinsic foot muscles [11–17], resulting in the decreased strength of the musculature of the foot/ankle complex and affecting both extensor and flexor muscles [18].

In addition to plantar ulcers and DPN, type 2 DM is associated with changes in joint collagen [19] that compromise patients’ physical functions related to daily living activities [20]. These functional deficits are due to aging joint collagen; decreased proteoglycans; and altered collagen types in ligaments, cartilage, and synovial fluids, which increase the rigidity of joint structures [21]. These changes result from the non-enzymatic glycation of collagen [22], which results in the chronic accumulation of advanced glycation products [23, 24]. The increased stiffness of the skeletal muscle lead to an increased injury risk [25], decreased range of motion and impaired function [26, 27]. Such morphological alterations, found in patients with DM and DPN, lead to foot deformities that cause structural and functional changes to the foot region [28]. These changes are responsible for increased pressure in certain plantar areas, a factor directly related to the formation of plantar ulcers [3, 7, 9, 11, 29, 30].

Within this context, some researchers have listed the prevention of ulcers as a priority in terms of the study of the diabetic foot [31, 32]. To optimize research into care strategies for those affected, the IWGDF established ulcer risk classification [5]. The classification consists of a stratification of people with DM into different categories of risk in terms of developing ulcers; a patient’s risk is determined through a clinical evaluation, the presence or absence of foot deformities, and the patient’s history of foot ulcers [5]. However, it should be noted that studies related to the prevention of plantar ulcers in people with DPN are ongoing and have had fewer positive findings [31] than studies related to the treatment of plantar ulcers.

When treating and preventing complications in DM patients with DPN, it is crucial to consider the strength levels of the patients’ lower limbs, which are their most affected body areas [33]. Reductions in lower limb strength can lead to negative consequences, such as reduced mobility and function, which affect walking, running, stair climbing, and other daily living activities [33]. Patients can lose the ability to perform physical exercises important for metabolic control, physical capacity maintenance, and quality of life [34]. Furthermore, the reduction of the foot–ankle strength are related to fall incidences in people with DM [35]. Thus, determining effective prevention strategies for such patients involves the characterization of the population in question in regards to deficits in sensitivity, musculoskeletal properties and mechanics; and function, the objective of this study was to investigate the foot function, strength, and health of people with DM, with or without DPN, based on the different ulcer risk classifications of the IWGDF.

## Methods

This cross-sectional study was conducted in the National Association of Assistance to Diabetes from May 2016 to December 2016 using a random series of recruitment and evaluations performed within this period. It was approved by an ethics committee (Comitê de Ética em Pesquisa da Faculdade de Medicina da Universidade de São Paulo, protocol 075/16). In sum, 240 people who were diagnosed with either type 1 or 2 DM, with or without DPN, were invited to participate. From the 240 subjects, 168 did not consent or did not meet the inclusion criteria, which were: participants aged between 40 and 75 years old, diagnosed with type 1 or type 2 DM and absence of active plantar ulcers, major vascular complications and severe nephropathy. The age range specified as an inclusion criteria was established considering that (1) our sample consisted of type 2 DM patients, (2) whose diagnosis occurred around 40-45 years old and (3) DPN is a chronic complication that starts approximately 10 to 15 years after the first diagnosis of DM [36]. In addition, the maximum age of 75 years was established because significant changes in muscle strength and tropism are present in older people and could have biased our results.

Patients were excluded if they had other neurological impairments, such as dementia; if they had other orthopedic impairments; if they were unable to give consistent information; and if they had active plantar ulcers, major vascular complications, severe retinopathy, or severe nephropathy. Although the IWGDF classifies DM patients into four categories [5], we have not included patients from Group 3, which comprised patients with DPN and history of foot ulcer or lower-extremity amputation (minor or major) or end-stage renal disease was not included in the study, first because of the nature of the recruitment setting that is a preventive care center and severe patients are not the target, second because if any minor or major amputation was present, it would refrain them to accomplish the strength assessment.

**Fig. 1:**
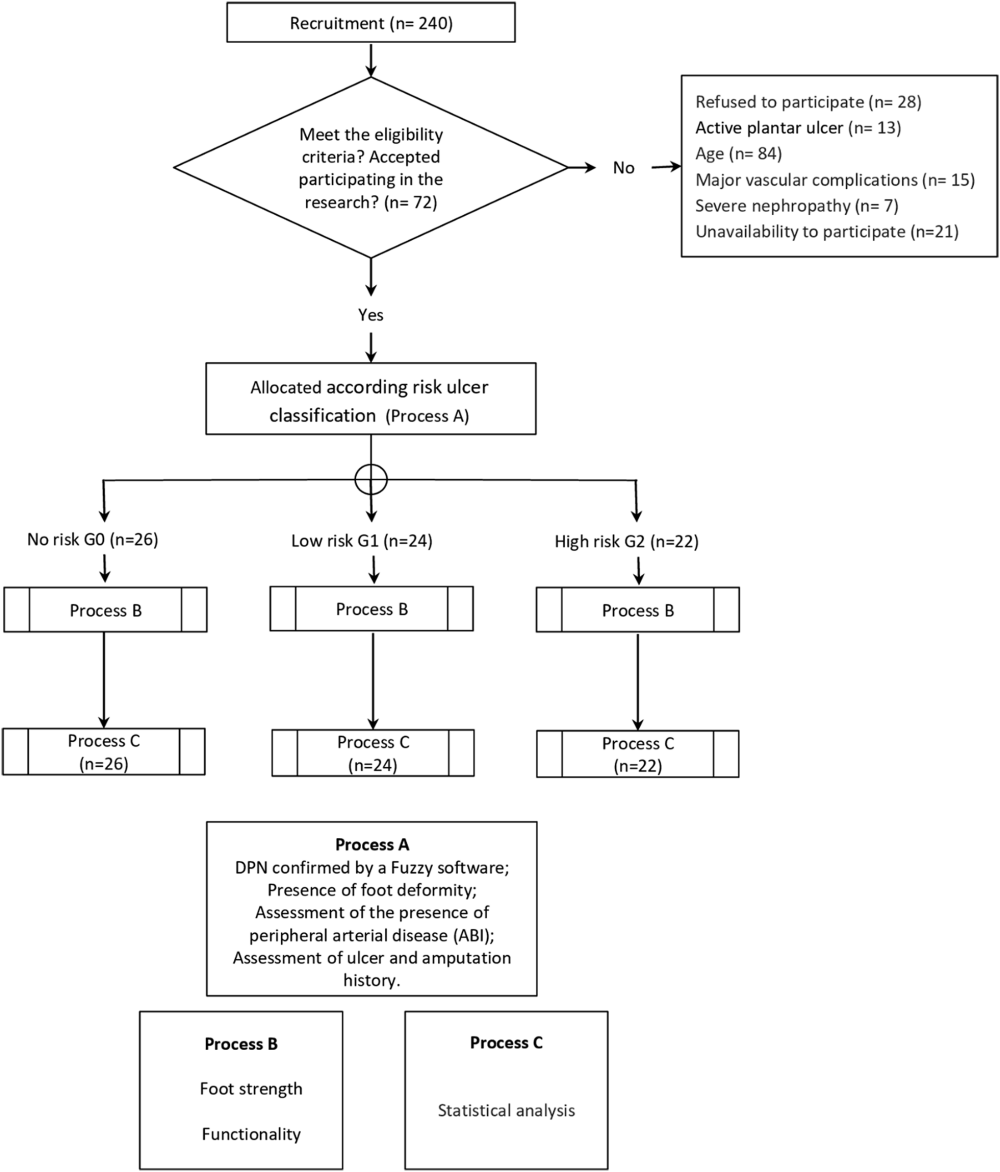
A flow chart of the assessments made at the ANAD in São Paulo

The final subject pool comprised 72 subjects, who were divided into three groups based on the ulcer risk classifications of the IWGDF [5] (Fig. 1): Group 0, which comprised patients without DPN; Group 1, which comprised patients with DPN; and Group 2, which comprised patients with DPN and with foot deformities and/or vascular disease.

To include and classify the subjects, DPN, vascular disease, and foot deformities were assessed. The subjects were screened for DPN using the fuzzy score software (http://www.usp.br/labimph/fuzzy/ingles/index.php). Fuzzy scoring is an rule-based expert system that supports the classification of DPN into different levels, based on severity [12, 37]. The system has an adequate accuracy with an Receiver Operating Characteristic ROC curve area = 0.91) for expert classifications [12]. The model combines the results of tactile sensitivity evaluations (conducted using a 10 g monofilament); the results of vibratory sensitivity evaluations (conducted using a tuning fork); and symptoms of DPN. Numerical values ranging from zero to 10 are produced that can be sorted into different classes. For this study, values above 2.5 were considered; such values indicated that DPN was present.

In addition, peripheral arterial disease was determined and classified using an ankle-brachial index; values of less than 0.5 indicated the presence of severe vascular disease; values ranging from 0.5 to 0.9 indicated the presence of vascular disease; and values ranging from 0.9 to 1.2 were considered normal [38]. Finally, the presence of deformities was determined through visual inspections conducted by trained physiotherapists. The inspections determined the presence of claw toes, hammer toes, flat foot, cavus foot, callosities, and/or hallux valgus. The presence of only one deformity was necessary for the classification of deformed feet. The subjects’ demographic, anthropometric, and clinical characteristics, and the subjects’ ulcer risk classifications, are shown in Table 1.

**Table 1:** Mean and standard deviation and p-values of demographics, anthropometrics and clinical characteristics of the studied groups

	G0 (n = 26)	G1 (n = 24)	G2 (n = 23)	p
Age (years)	65.0 ± 13.9	63.0 ± 16.1	60.5 ± 11.6	0.522^1^
Males (%)	61.5	55.0	35.7	–
Body mass (kg)	73.0 ± 15.6	72.0 ± 11.9	67.5 ± 15.1	0.872^1^
Height (cm)	162.0 ± 11.4	164.0 ± 5.8	158.0 ± 10.8	0.883^1^
Body mass index (kg/m^2^)	32.5 ± 4.9	25.5 ± 3.6	27.9 ± 3.7	*0.001^1^
Diabetes diagnosis (years)	11.2 ± 7.7	15.9 ± 11.8	11.8 ± 9.1	0.234^1^
Presence of DPN (%)	0	100	100	
Tactile sensitivity loss [10 g monofilaments] (%)	0	100	100	
Vibratory sensitivity				
Absent (%)	0	20.7	41.4	0.073^2^
Reduced (%)	0	79.3	58.6	0.759^2^
Presence of peripheral vascular disease (%)	0	0	8.7	
Presence of foot deformities (%)				
Claw toes	0	0	52.0	
Hammer toes	0	0	13.0	
Flat foot	0	0	13.0	
Hallux valgus	0	0	22.0	

*Statistically significant difference

^1^p values for the ANOVA tests

^2^p values for the Chi-square tests

### Outcomes and assessments

An initial assessment was conducted to determine whether the subjects met the inclusion criteria. Then, a second assessment was conducted using the fuzzy score software to evaluate the presence and classify the severity of DPN in patients. The presence of neuropathy was classified by symptoms using the Michigan Neuropathy Screening instrument. The greater the score, the greater the symptoms amount and thus the DPN severity (Table 1). The assessment protocol also comprised two subsequent evaluations: the foot health status assessment based on the Foot Health Status Questionnarie (FHSQ-BR) [39], which was completed in a quiet room by all participants, and measurements of the subjects’ hallux and toe strengths, conducted using the emed q-100 pressure platform (Novel, Munich, Germany) (Fig. 1).

#### Foot strength assessments

The subjects’ isometric foot strength was measured using the emed pressure platform; this method is a reliable way to assess foot muscle strength with Intraclass Correlation Coefficient between assessment sessions (ICC) greater than 0.92 [40]. Each subject stood and pushed down on the pressure platform three times, as hard as possible, with his or her hallux and toes from one side randomly chosen before his/her first trial. The mean value of all trials from one foot both was used for statistical purposes. The maximum force under the patient’s hallux and toes, normalized by patient’s bodyweight, were the outcomes of this measurement.

#### Foot health and function assessments

This study used a Brazilian Portuguese version of an (FHSQ-BR) that was translated and validated [39]. Section I evaluated foot health using four domains: foot pain, foot function, footwear, and general foot health. Section II referred to general health, and Section III collected general demographic data. Only scores from Section I were used because Section II examined general health. Each domain in Section I could result in a score ranging from zero to 100 points, where 100 was the best condition and zero was the worst condition. The scores were calculated using version 1.03 of FHSQ software (Care Quest, Queensland, Australia).

### Statistical analyses

A sample-size calculation was performed using version 3.1 of the software G*Power [41]. For this calculation, it was assumed: a type I error (α) of 5%, a power of 85%, a large effect size with an *f* of 0.40, 3 groups, and a statistical design of one-way ANOVA. A number of subjects (72) were reached. For an inferential statistical analysis, continuous data were presented as means and standard deviations, categorical data are presented in percentage, comparisons among groups (G0, G1, and G2) for the continuous data were made using one-way ANOVAs for each variable analyzed, and Chi-square tests were used for other categorical data. These analyses were made using version 12 of the software Statistica. Significant differences were considered with an α of 5%.

## Results

**Fig. 2:**
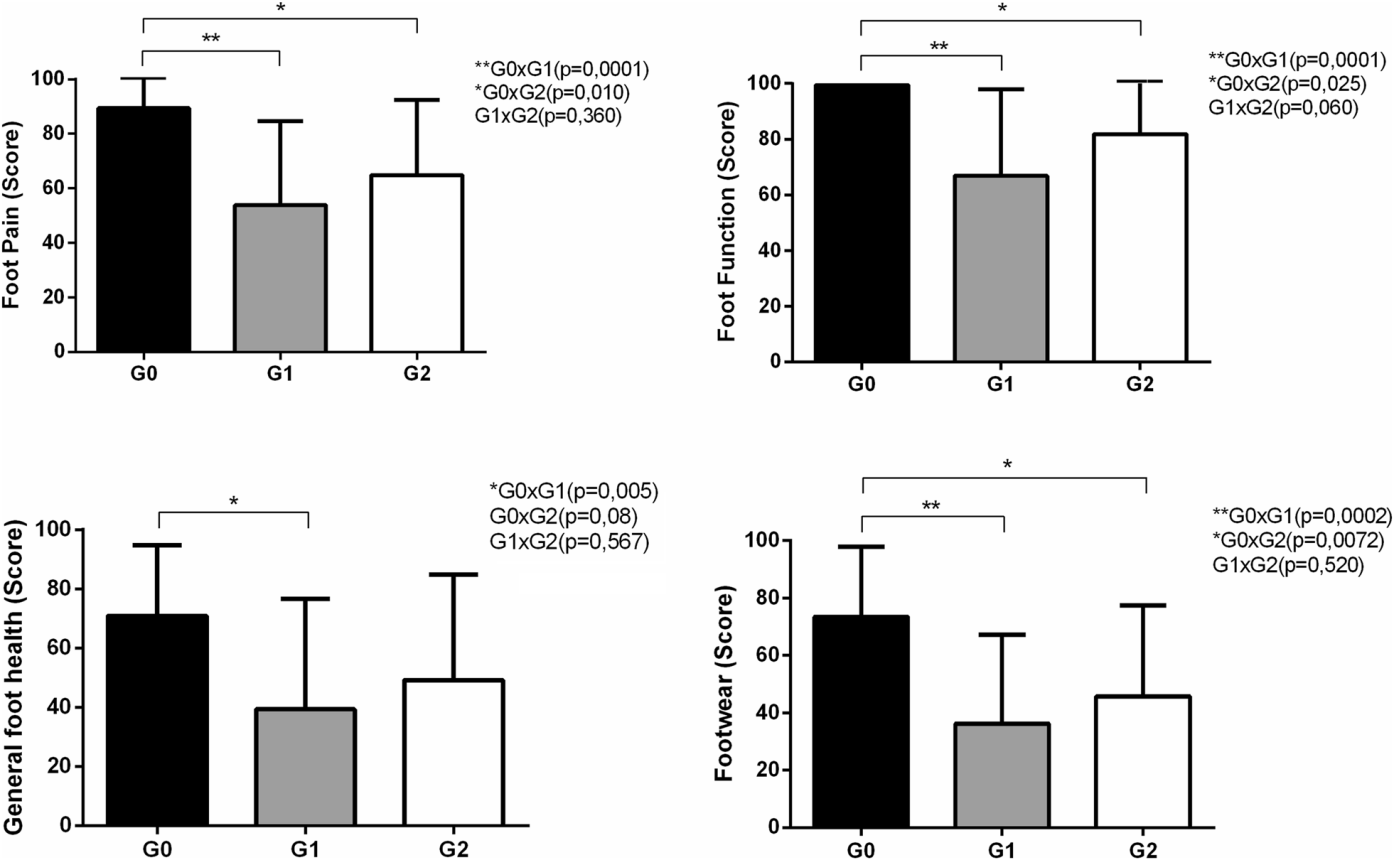
Mean and standard deviation scores of the FHSQ questionnaire and comparisons among groups

Regarding foot health status and foot function, there were moderate differences between the groups, especially between G0 that had significantly higher scores in all domains in comparison to the other groups (Fig. 2). G1 and G2 had smaller numerical mean differences than with G0, but G1 had smaller scores in all categories. Moreover, significant differences were found in the following domains: foot pain (for G0 × G1 and G0 × G2), foot function (for G0 × G1 and G0 × G2), general foot health (for G0 × G1), and footwear (for G0 × G1 and G0 × G2) (Fig. 2).

**Fig. 3:**
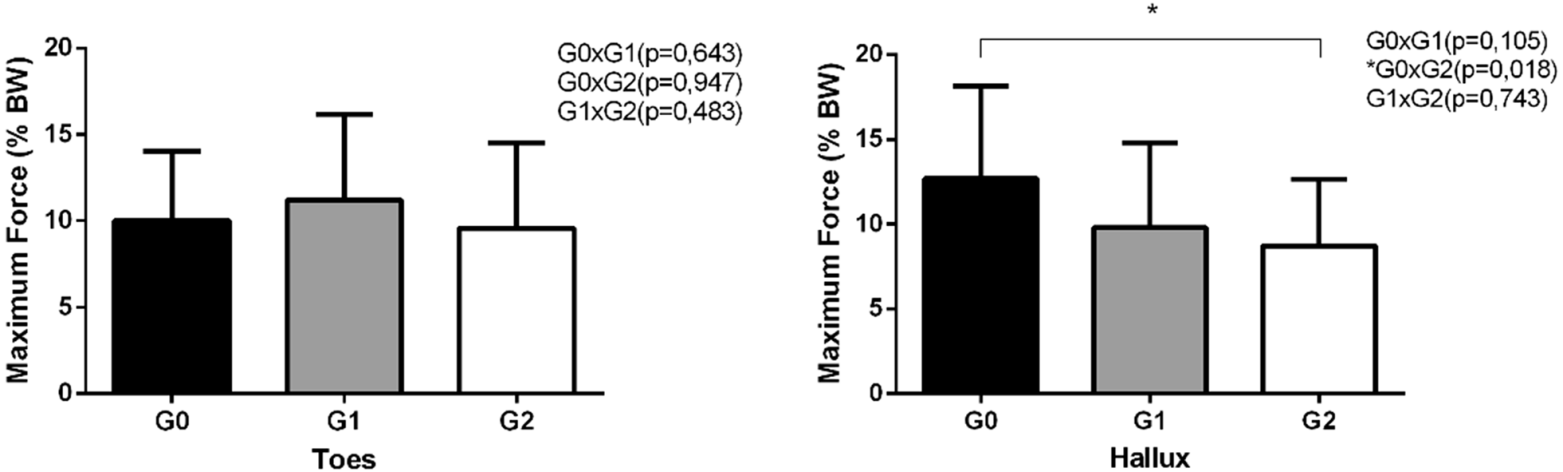
Comparison of the mean of the toes and hallux strength among different categories of ulcer risk

The patients in G1 and G2 had smaller hallux strength values than the patients in G0; these variables indicated ulcer formation risks. However, there were no statistical differences between the ulcer risk groups in terms of toe strength (*p* was 0.05). The hallux strengths of G2 patients were significantly lower than the hallux strengths of G0 patients (Fig. 3). Moreover, the hallux strengths decreased as ulcer risks increased.

In summary, moderate differences were found between G0 and G1 and G2 for foot pain, foot function, general foot health, and footwear. There was a small but significant difference between G0 and G2 in regards to hallux strength. No significant differences were found between G1 and G2 for any of the variables (Table 2).

**Table 2:** Mean difference between groups and 95% confidence interval (CI) for all variables

	G0 × G1 mean diff (95% CI)	G0 × G2 mean diff (95% CI)	G1 × G2 mean diff (95% CI)
Foot pain (score)	35.4 (16.3 to 54.6)	24.5 (4.9 to 44.0)	− 10.9 (− 30.0 to 8.1)
Foot function (score)	32.5 (17.0 to 48.1)	17.6 (1.7 to 33.4)	− 14.9 (− 30.5 to 0.5)
General foot health (score)	31.5 (8.1 to 54.8)	21.5 (− 2.2 to 45.4)	− 9.9 (− 33.2 to 13.4)
Footwear (score)	37.2 (16.4 to 57.9)	27.7 (96.5 to 48.9)	− 9.4 (− 30.2 to 11.2)
Toes (% BW)	− 1.1 (− 4.3 to 1.9)	0.4 (− 2.8 to 3.6)	1.6 (− 1.7 to 4.9)
Hallux (% BW)	2.8 (− 0.4 to 6.2)	3.9 (0.5 to 7.3)	1.0 (− 2.4 to 4.5)

*BW* body weight

## Discussion

Determining effective prevention strategies for people with DM and DPN involves a comprehensive description of the population in question regarding deficits in sensitivity, musculoskeletal properties, and function. This study investigated the foot health, toe and hallux strength levels, and foot function of people with DM based on the IWGDF’s ulcer risk classifications [5]. Significant differences were observed in the function levels of the different classifications, and G2 had significantly lower hallux strengths than G0.

An interesting finding is that G1, which comprised patients with DPN, had the highest functional deficits, and G2, which comprised patients with DPN and foot deformities and/or vascular disease, presented the lowest strength of the hallux flexor and toes flexor muscles. The DPN severity may be a determining factor for which G1 presents the greatest impairments in function. Patients with foot deformities (G2) presented lower levels of muscle strength, but it is still not clear in the literature whether a decrease in muscle strength may be responsible for the development of foot deformities or the result [42]. The fact that G2 is composed of younger patients with foot deformities and lower strength levels, and G0 is composed of older patients without DPN, may be somewhat specific to the population of this study. Although the mean age of the groups were not statistically different and could not be considered a bias in the results, the self-care strategies adopted by each patient, that unfortunately could not be controlled, may have influenced the function and strength level within groups.

The differences observed between groups in function (i.e., foot function, foot pain, general foot health, and footwear) showed that G0 had better outcomes than G1 and G2. Several studies support that patients with DPN (G1 and G2) have larger functional losses [43–45]. Some studies found that patients with DPN had higher levels of pain than patients without DPN [46–48]. It has also been shown that people with DM and DPN presented a decreased muscle strength and sensitivity that are associated to impairments in everyday locomotor activities [33]; such as reduced gait velocities, muscle activity alteration [49], lower limb kinematic pattern changes [50], and balance impairments [51–53]; however causality is difficult to specify. DPN patients also present loss of muscle function [54] by a manual muscle testing that may lead to functional losses. All these functional impairments made it difficult for these patients to perform daily living activities, can compromise the patient’s physical and mental health, and negatively impact his or her quality of life [55].

G1 and G2 patients had lower hallux strengths than G0 patients. A possible reason for this is that the flexor hallucis longus and brevis have a smaller cross-sectional area than the flexor digitorum longus and brevis [56]. Thus, a minimal atrophy can lead to a significant reduction in force. Unlike the flexor muscle of the toes due the latter’s greater cross-sectional area and, consequently, greater force generation. Although there has been no research similar to this study involving the foot strength of patients with different ulcer risk classifications, some studies have shown that the deterioration of the intrinsic foot muscles associated with low ranges of ankle motion might be a primary factor in the development of foot deformities [28]. Furthermore, toes deformity would put the intrinsic and extrinsic foot muscles at a poorer mechanical advantage, changing their fascicle length and lever arms, thus less force would be generated [57], just like we found in this study for strength levels in G2 (patients with foot deformities) compared to G0. Either way, as a cross sectional study, causality cannot be assessed to determine the causes of the lower strength levels in G2.

In people with DPN, Andersen et al. [33, 58] and Bus et al. [42] found atrophied foot muscles and increasing severity of DPN was associated with low ankle muscle strength that was proportional to muscular atrophy levels. A similar study found that decreased ankle strength was progressive and was more pronounced in older age groups than in younger age groups [59]. These findings corroborate this study’s findings, including the observations that G2 had lower hallux strength than G0 and G1.

In a prior study, the atrophy of diabetic foot muscles was verified in a subclinical stage of DPN before the development of clinical DPN symptoms [60]. This showed that predicting/assessing foot strength and function of individuals with DM, and following such predictions with early interventions, could be a viable strategy to prevent the accelerated loss of muscle strength and to reduce the risk of developing ulcers in patients. This strategy could contribute to a better quality of life for these individuals.

This study had some limitations; first, the physical activity levels and the rehabilitation practices of the subjects could not be controlled. Second, there was no control group, and this prevented further conclusions regarding the consequences of foot function, foot strength, DM, and DPN progression. Finally, we emphasize that G3, defined by the IWGDF risk stratification system, was not included in this study due to the availability of these type of patient in the study setting, limiting our conclusions on muscle strength and foot function in more severe patients. Further studies should account for that when recruiting patients in different settings to include a broader range of risk categories.

Future studies should overcome these limitations; that is, they should reduce the heterogeneity of groups and include control groups. Further studies should also aim to improve the IWGDF’s ulcer risk classifications by considering analyses of the foot strength levels and foot function of people with DM and DPN.

## Conclusion

The foot function, hallux strengths, and foot health of people with DM and DPN differ by ulcer risk classification. These characteristics must be considered when evaluating and developing treatments and preventative strategies for such patients. In particular, patients with risk 2 classifications are more severely impaired in regards to foot strength and foot function than other patients. Early assessments of lower limb musculoskeletal deficits could potentially prevent changes to functions that occur with the progression of DM.

## Data Availability

All personal data from potential or enrolled patients will be maintained confidential before, during and after the study by encoding participant’s name. All data access and storage are in keeping with National Health and Medical Research Council guidelines, as approved.

## Abbreviations

IWGDF: International Working Group on the Diabetic Foot
CNPq: National Council for Scientific and Technological Development
CAPES: Agency Coordination of Improvement of Higher Education Personnel
DM: diabetes mellitus
DPN: diabetic polyneuropathy
G0: Group 0
G1: Group 1
G2: Group 2
FHSQ BR: Brazilian version of Foot Health Status Questionnaire
ANAD: National Association of Assistance to Diabetics
ICC: Intraclass Correlation Coefficient
BW: body weight
